# Effects of Rust Fungus and Aphid Infestation on Plant Height and Seed Production of *Impatiens parviflora* DC.

**DOI:** 10.3390/plants15172655

**Published:** 2026-08-29

**Authors:** Péter Csontos, Damian Chmura, Károly Penksza, Zsuzsanna Angyal, András Albert Halbritter, Orsolya Pintér, Zsófia Kovács, Tibor Kalapos, Júlia Tamás

**Affiliations:** 1 Institute for Soil Science, Centre for Agricultural Research, Hungarian Research Network, Fehérvári út 132-144., H-1116 Budapest, Hungary; csontos.peter@atk.hun-ren.hu (P.C.); kovacs.zsofi@atk.hun-ren.hu (Z.K.); 2 Centre for Environmental Science, Faculty of Science, ELTE Eötvös Loránd University, Pázmány P. stny. 1/C, H-1117 Budapest, Hungary; angyal.zsuzsanna@ttk.elte.hu; 3 Institute of Environmental Protection and Engineering, University of Bielsko-Biała, 2 Willowa Str., PL-43-309 Bielsko-Biała, Poland; dchmura@ubb.edu.pl; 4 Department of Botany, Institute of Agronomy, Hungarian University of Agriculture and Life Sciences, Páter K. u. 1., H-2100 Gödöllő, Hungary; 5 Department of Elementary and Pre-School Pedagogy, István Nemeskürty Faculty of Teacher Training, Ludovika University of Public Service, Ludovika tér 2., H-1083 Budapest, Hungary; halbritter.andras.albert@uni-nke.hu; 6 Department of Integrated Plant Protection, Institute of Plant Protection, Hungarian University of Agriculture and Life Sciences, Páter K. u. 1., H-2100 Gödöllő, Hungary; pinter.orsolya@uni-mate.hu; 7 Department of Plant Systematics, Ecology and Theoretical Biology, Faculty of Science, Institute of Biology, ELTE Eötvös Loránd University, Pázmány P. stny 1/C, H-1117 Budapest, Hungary; tibor.kalapos@ttk.elte.hu; 8 Department of Botany, Hungarian Natural History Museum, Hungarian National Museum Public Collection Centre, Könyves K. krt. 40., H-1089 Budapest, Hungary; tamasjuli9@gmail.com

**Keywords:** biological control, *Impatientinum asiaticum*, invasive neophyte, plant herbivore interaction, *Puccinia komarovii*, shoot height, seed mass, seed number, small balsam

## Abstract

The effects of the rust fungus *Puccinia komarovii* Tranzschel and the aphid *Impatientinum asiaticum* Nevsky 1929 on plant height, seed number per capsule, and seed mass were investigated in eight small balsam (*Impatiens parviflora* DC.) stands in Hungary and one in Poland. Two stands were infected by rust, five by aphids, and two were healthy. The lowest average plant height was 37.5 cm and the highest 94.7 cm, both measured in aphid-infested stands. Examining the stands separately, no relationship appeared with the type of damage. For data pooled across stands, differences were significant: rust-infected plants were the tallest, healthy shoots were the shortest, while aphid-infected plants fell in between. Mean seed number per capsule was the lowest (1.53) in the pest-free Nagybörzsöny stand, and the highest (2.33) in the aphid-infested Bielsko-Biała stand. In pairwise comparison of stands, average seed number did not differ in most cases. Significant positive correlation was found between average plant height and average seed number per capsule. Seeds were the heaviest in the healthy stand, whereas they were lighter in rust-infected and aphid-damaged stands. Regarding the applicability of the studied pests as biological control agents, the results showed that both pests reduce seed mass but not overall performance of the host plant.

## 1. Introduction

The distribution area of some alien species that have been previously introduced to the Hungarian flora is still showing continuous expansion [1,2,3,4]. In the case of such increasingly invasive species—which include the small balsam (*Impatiens parviflora* DC.)—it is particularly important to gain detailed knowledge about their life history and ecological relationships, as this may be useful in controlling them.

Small balsam is native to Central Asia, and its first subspontaneous occurrence in Europe was recorded in 1831 near the Geneva Botanical Garden [5]. Subsequently, in the initial stages of its invasion, it was observed primarily as a garden escapee in various cities [6]. It now occurs in almost all European countries [7,8,9,10,11,12] and has also been introduced to North America [13]. Its distribution mainly occurs in the understory of closed forests [8], and its detection, therefore, requires intensive field work. Remote sensing methods, which have been successfully used in the monitoring of other invasive herb species inhabiting open, non-woody habitats [14,15,16], are not practically applicable to *I. parviflora*, the stands of which are generally shaded by dense tree canopy.

**Figure 1:**
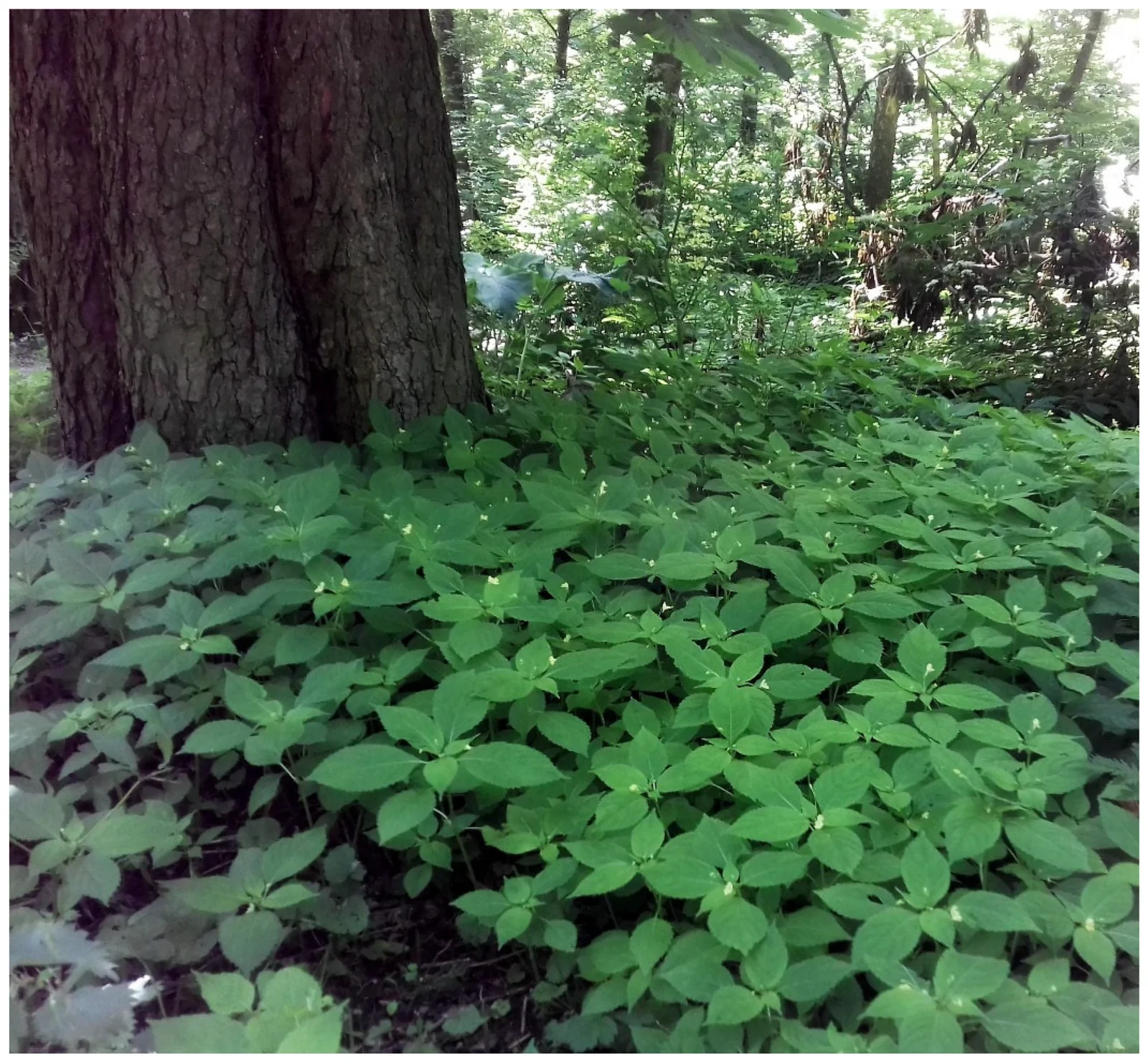
Dense stand of *Impatiens parviflora* at a riverine woodland in Hungary (photo by P. Csontos).

**Figure 2:**
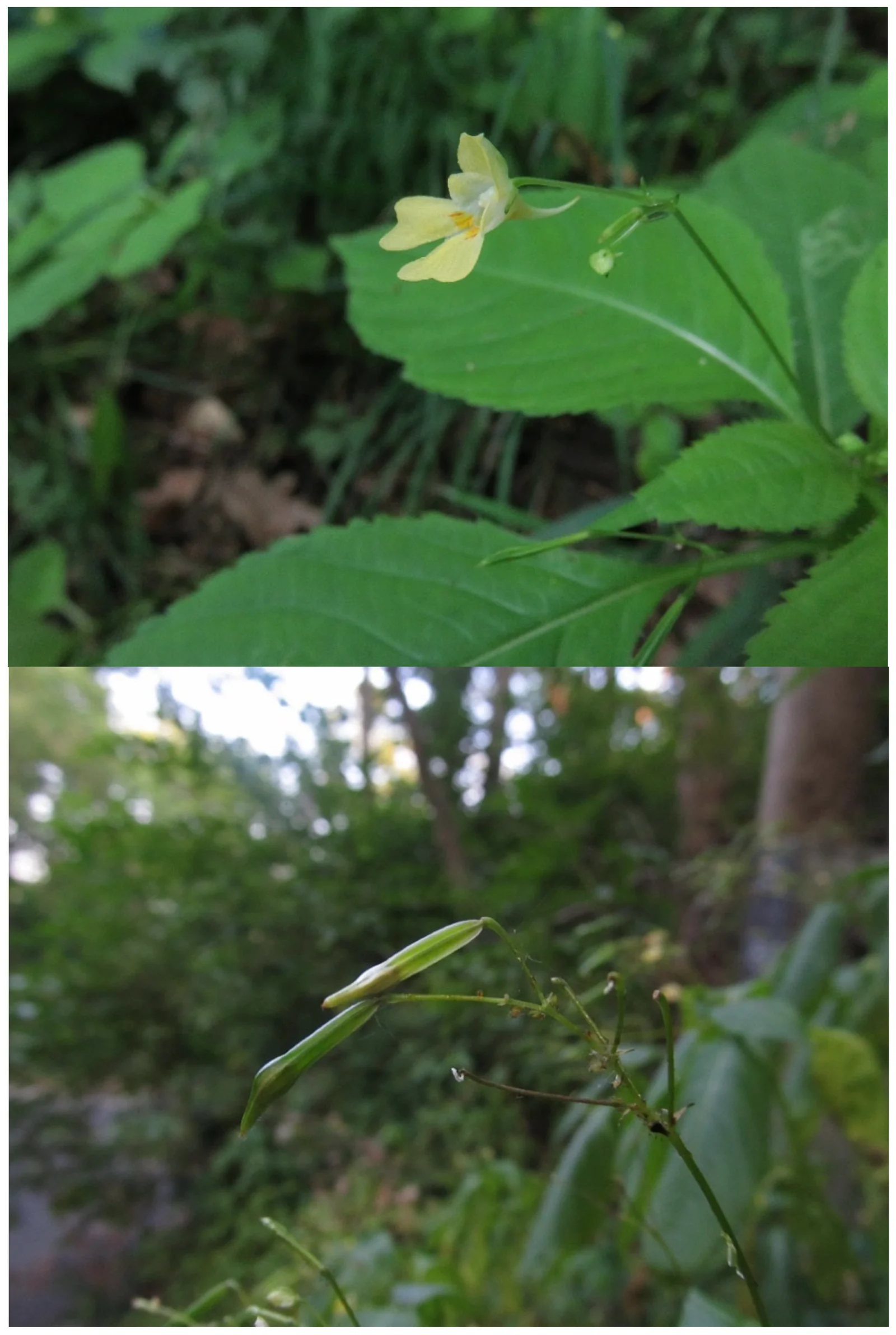
Zygomorphic flower (**upper picture**) and two one-seeded capsules (**lower picture**) of *Impatiens parviflora* growing in a healthy stand in Hungary (photos by P. Csontos).

The success of invasive plant species in new geographic regions is often attributed to the absence of natural enemies that keep these species under control in their original habitats [17,18]. Small balsam is now quite common along the riverbanks and in the humid or mesophytic forests of Hungary, and its appearance in these places is usually abundant [19,20] (Figure 1 and Figure 2).

The natural diseases and pests of *I. parviflora*, the rust fungus *Puccinia komarovii* Tranzschel ex P. Syd & Syd. (Basidiomycota, Pucciniaceae) and the aphid *Impatientinum asiaticum* Nevsky, 1929 (Arthropoda, Aphididae), have also become known from their Hungarian populations [21]. Host-specific diseases and pests of invasive species are often used in biological control [22], although these interventions are not always successful [23]. However, a variety of the rust fungus species (*Puccinia komarovii* var. *glandulifera*) is already being used in biological control of *Impatiens glandulifera* Royle in Great Britain [24,25], where it has shown great efficiency in semi-natural conditions [26].

Regarding the aphid, *I. asiaticum*, a Bulgarian study mentions it as a possible biocontrol agent, based on the fact that this species feeds on invasive Himalayan balsam plants, but no controlled host specificity and efficacy studies have been conducted to consider *I. asiaticum* as an actual biocontrol agent [27]. On a broader level, it should be noted that another aphid species, *Aphis chloris*, has been used in Australia to control invasive *Hypericum perforatum* [28]. However, there are few known examples of aphids being used as biological control agents.

In this paper, we seek to answer the question of whether host-specific diseases and pests, namely infection by *P. komarovii* and damage by the aphid species *I. asiaticum*, can be exploited as biological control agents, i.e., do they cause a decrease in vitality or decline in the development and reproductive success of the host plant in *I. parviflora* stands?

## 2. Results

**Figure 3:**
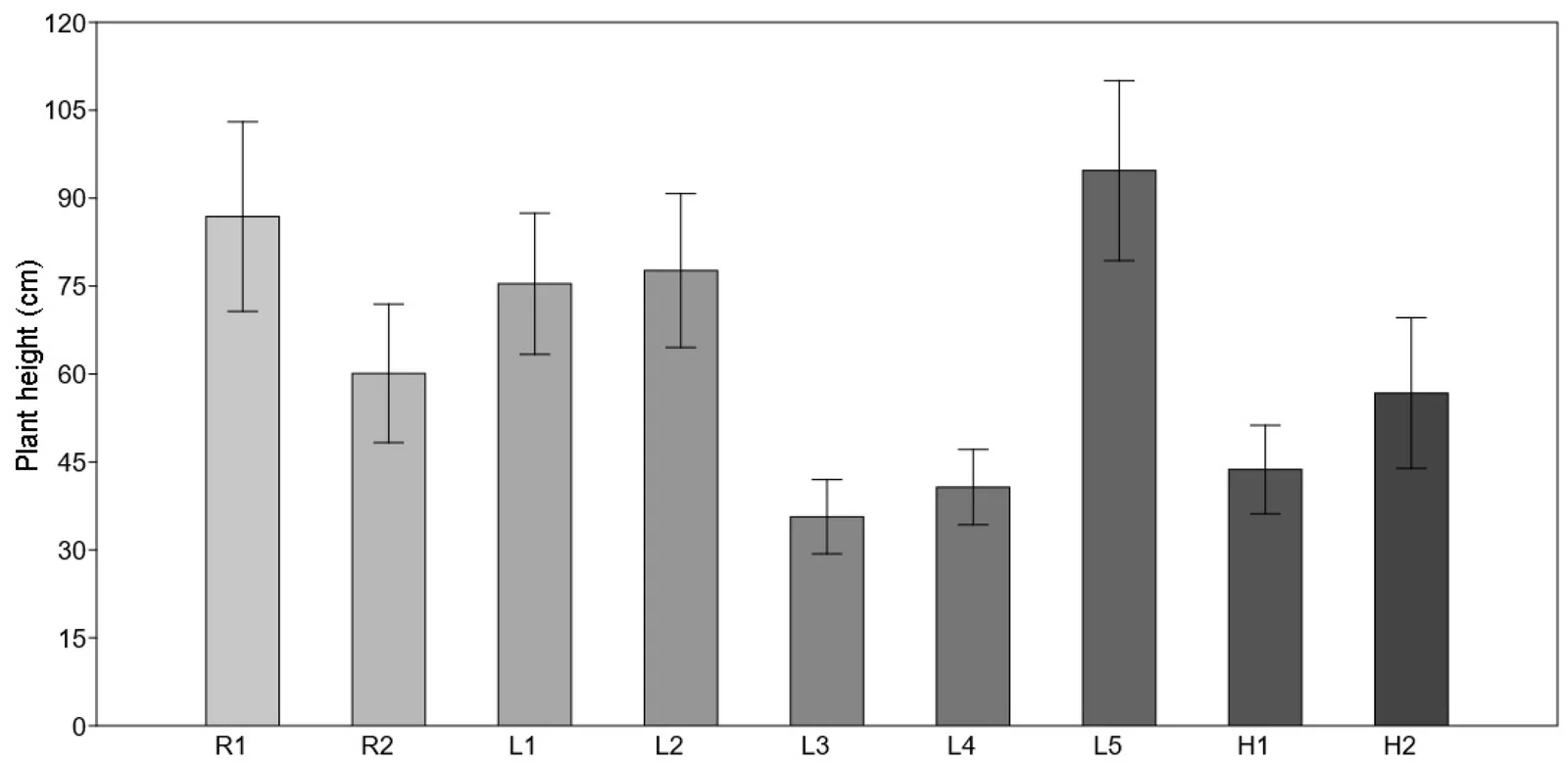
The average height of individuals of *Impatiens parviflora* in infected or pest-exposed and healthy stands (*n* = 20). R1–2: infected by rust fungus *Puccinia komarovii*; L1–5: damaged by aphid *Impatientinum asiaticum*; H1–2: healthy stands. The bars fitted to the columns indicate the standard deviation (*n* = 20).

The tallest plant height, averaging 94.7 cm, was found in the stand surveyed near Bielsko-Biała (L5, Poland), while the individuals of the stand at Kis-Sváb-hegy (L3, Budapest, Hungary) were the lowest, with an average height of 35.7 cm. Both extreme average values were found among the stands damaged by aphids (Figure 3) and, similarly, the smallest and tallest specimens measured among all examined individuals were also found in this group (25 cm and 126 cm, respectively). The two stands infected with rust fungus were slightly taller than the healthy stands (Figure 3), but significant differences were restricted to the R1 stand. Of the 36 possible pairings between the nine stands, significant differences were found in 19 cases (*p* < 0.05), while in 17 cases the difference was not significant (*p* > 0.05). In addition, the stand pairs showing significant and non-significant differences occurred mixedly among the health status groups. Overall, it can be said that the stands with three different health statuses (rust- or aphid-infected, and pest-free), if all nine are examined independently, did not form clear groups based on the height of their individuals.

When data from stands with the same health status were combined, all three types were significantly different in terms of average plant height of *I. parviflora* (Table 1). The rust-infected specimens were the tallest, followed by the aphid-infected ones, while the healthy plants were the shortest.

**Figure 4:**
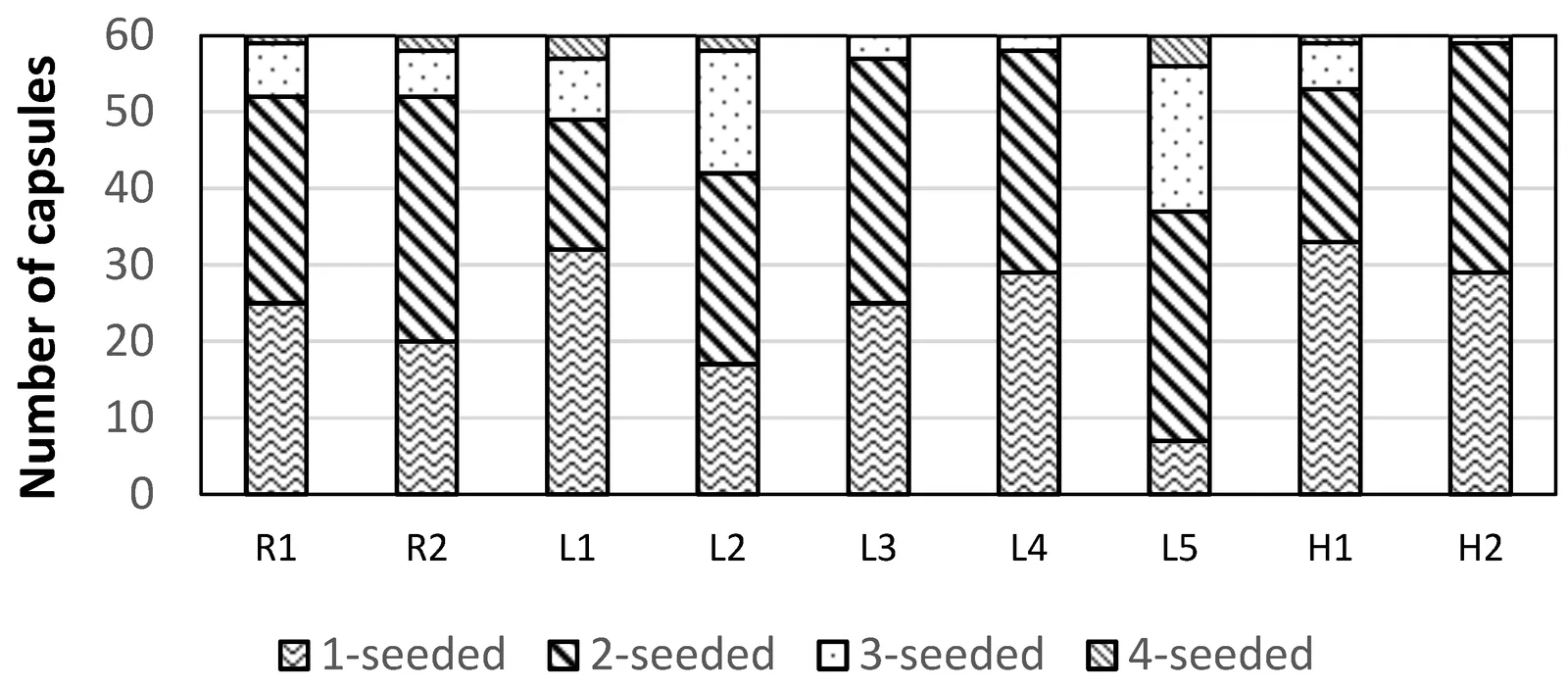
Number of capsules according to their seed content in nine stands of *Impatiens parviflora*, based on 60 capsules per stand. R1–2: infected by rust fungus *Puccinia komarovii*; L1–5: damaged by aphid *Impatientinum asiaticum*; H1–2: healthy stands.

When we examined the number of seeds per capsules, we did not find any five-seeded capsules—which would have been the maximum possible [29]—and in some stands no four-seeded capsules were found either. Overall, however, capsules with one to four seeds were present in all three stand types (Figure 4). The greatest variability in seed number per capsule (similarly to the case of plant height) was observed in aphid-damaged stands.

The results of the statistical analysis performed on the seed number per capsule data are summarized in Table 2. The lowest average seed number was found in the pest-free Nagybörzsöny (H2) stand (1.53 seeds/capsule), and the highest was observed in the aphid-infested (L5) stand in Bielsko-Biała (2.33 seeds/capsule). The number of seeds per capsule in the L5 stand was significantly higher than that of all other stands except the L2 stand (it should be noted that the height of the individuals was also the tallest in the L5 stand). Furthermore, the aphid-damaged stand at Normafa (L2) showed significant differences compared to the three populations with very low average numbers of seeds per capsule, which were the stands surveyed near Nagybörzsöny (H2; 1.53 seeds/capsule), Budakeszi (L4; 1.55 seeds/capsule) and Vámosszabadi (H1; 1.58 seeds/capsule) (Table 2). In the other pairings of the stands, the number of seeds per capsule data did not differ significantly.

**Figure 5:**
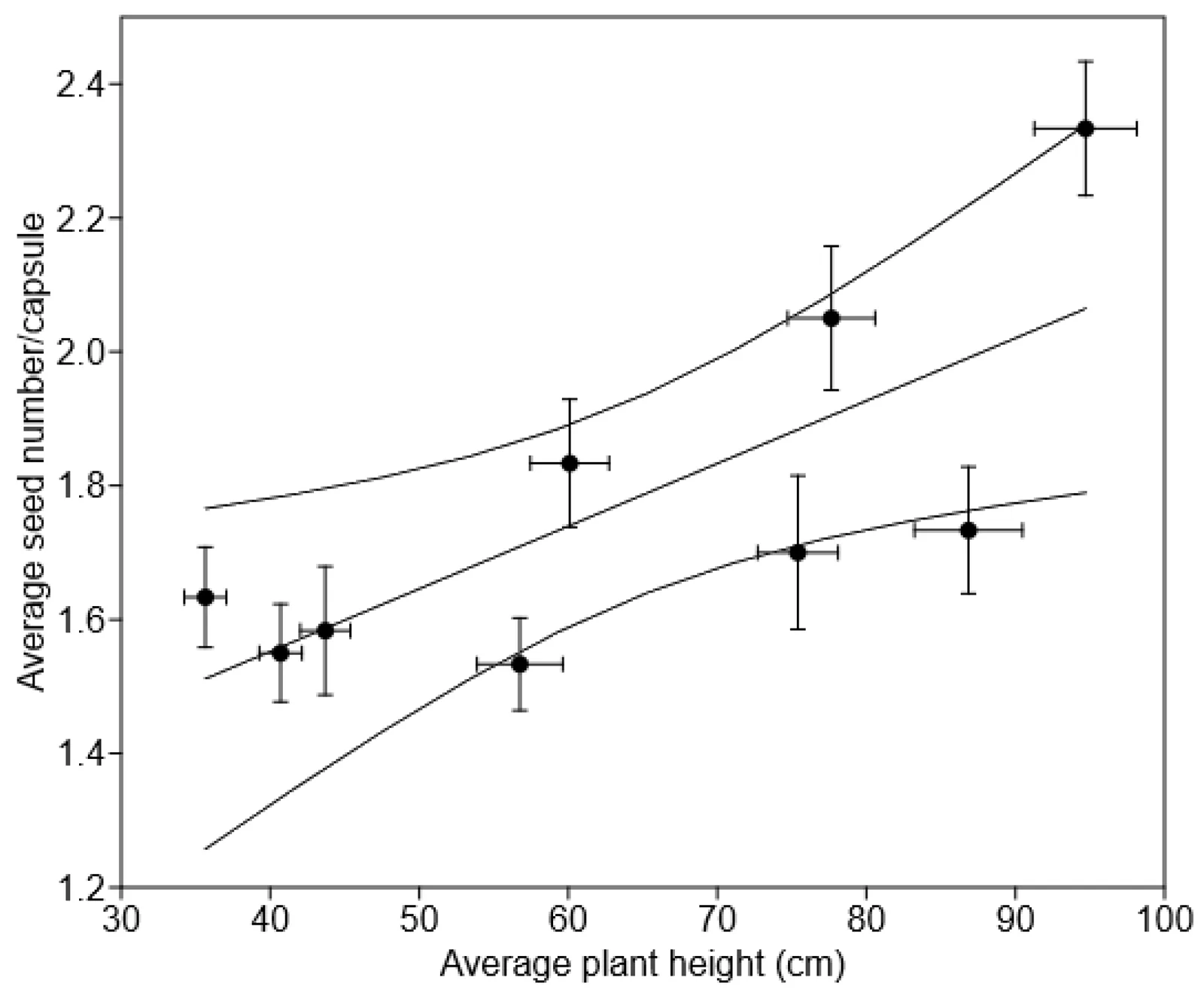
Linear regression between average plant height (*n* = 20) and average seed number per capsule (*n* = 60), with 95% confidence interval indicated, based on nine stands of *Impatiens parviflora* (*r* = 0.7485). Bars indicate ±1 SE.

The relationship between the average plant heights and the average number of seeds per capsule in the stands is shown in Figure 5. The correlation coefficient (r) of linear regression was 0.7485, and the slope of the line deviated significantly from zero (*p* = 0.0203).

**Figure 6:**
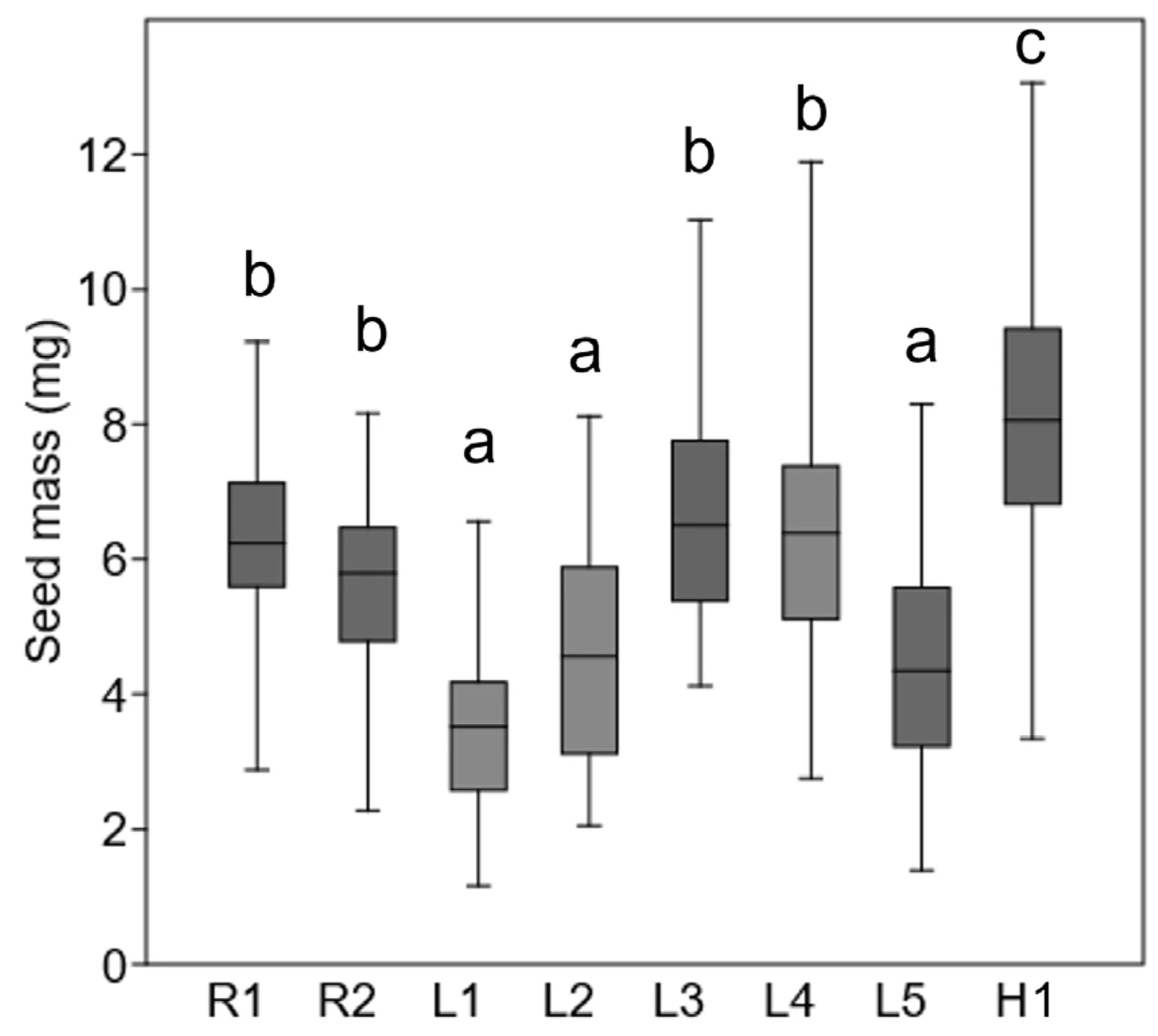
Box and whisker plots of seed masses (*n* = 70) of *Impatiens parviflora* in healthy (H1) and pest-exposed (R1–2: infected by rust fungus *Puccinia komarovii*; L1–5: damaged by aphid *Impatientinum asiaticum*) stands. The minimum, first quartile, median, third quartile, and maximum values are highlighted. Different letters above the boxes indicate significant differences among stands.

The results of the statistical comparison of the seed mass data among stands using the Kruskal–Wallis test and subsequent Dunn’s test are shown in Figure 6. The healthy stand (H1) was characterized by the heaviest seed mass, and it differed significantly from seed masses of all pest-exposed stands. Seed masses of the two rust-infected stands fell into the same statistical group; however, within aphid-damaged stands, wider variability of seed mass was detected.

## 3. Discussion

The height of adult individuals of *I. parviflora* is given by Coombe [29] as 20 cm to 150 cm, by Chmura [30] as 4 cm to 152 cm, and by Csiszár and Bartha [20] as 10 cm to 150 cm. The individual height data observed in the present study are consistent with these data. The literature also reports that the average height of individuals of *I. parviflora* populations in different habitats can differ significantly [6,31,32]. In our case, this was clearly demonstrated in the five stands infested by aphids. Although aphid-infested stands were taller on average than uninfested stands, plant height varied considerably among infested sites. This suggests that habitat conditions probably modified the growth of *I. parviflora* to a greater extent than aphid infestation alone. Resource-rich habitats may allow plants to compensate for herbivore damage through enhanced growth, whereas resource limitation may constrain such compensatory responses [33,34,35]. However, when samples of stands belonging to the same category were treated together, the differences that had previously remained hidden became detectable due to the increased sample size. This also points out that it is worth working with a larger sample size in further studies.

The greatest individual height observed in rust-infected stands may be related to the fact that *P. komarovii* infection can cause shoot and hypocotyl elongation, as has been previously shown for *I. parviflora* individuals [36,37] and, regarding the hypocotyl stem only, for a related species, *I. glandulifera* [38]. Based on the theory of evolution, a possible explanation for the development of this phenomenon is that the effect of a rust fungus on shoot elongation may result in a better dispersal of the spores of such fungi. This is because the spores of the fungus can be carried by the wind from the higher leaves of elongated host plants more easily and over greater distances, so that rust variants with this trait can spread more widely. However, severe rust infection might have a negative effect as it resulted in stunting and death of individuals in stands in Slovakia [36,39].

As for the higher average height of aphid-damaged stands than healthy ones, it is supposed that plants in stands with more humid habitats which are favorable to aphid infestation generally grew taller, and under such conditions, their leaves also have a softer tissue structure than those of specimens growing in drier conditions. This assumption is consistent with general research results examining the relationship between leaf blade thickness and habitat water availability, although the relationship could not be demonstrated for some species [40,41]. Therefore, we suppose that softer leaves attract aphids to such *I. parviflora* stands, because they can feed more easily on them [42]. Additionally, the results reported in the literature indicate that aphids may preferentially colonize more vigorous host plants, resulting in a positive association between infestation and plant height without implying that infestation enhances growth [43,44]. Consequently, the observed pattern should be interpreted as an association rather than evidence that aphid infestation increases plant height.

The average values of the number of seeds per capsule varied between 1.53 and 2.33 in the studied stands. In two stands (L1 and H1) single-seeded capsules were the most frequent and in six other stands (R1, R2, L2, L3, L5 and H2) two-seeded capsules were the most frequent; in stand L4, single- and double-seeded capsules were equally common. This is a slightly higher fecundity than that observed in *I. parviflora* populations in five Polish forests, where in all cases single-seeded capsules dominated [6]. We did not examine the total seed yield per individual, because determining it would have required an extraordinary amount of field work due to the species’ prolonged seed dispersal and the dynamochor capsule type. Based on the literature data, in favorable habitats, in the case of 4–5-seeded capsules, the seed yield can reach 10,000 seeds per individual [29]; however, in the stands we examined, the seed yield was certainly significantly lower than this. None of the rust-infected stands showed significant differences from healthy stands in terms of seed number per capsule. Of the five aphid-infested stands, one (L5) showed a higher average seed count compared to both rust-infected and healthy stands; in another case (L2), we measured a higher seed count compared only to healthy stands, but the other three aphid-infested stands did not show significant differences. Therefore, it cannot be stated that the number of seeds per capsule in aphid-infested stands is consistently higher than in other stands. Furthermore, the multiple significant differences detected between individual stands within the group of aphid-infested stands, in terms of seed number per capsules, may actually indicate that the presence of aphids does not necessarily have a significant effect on this plant trait.

Based on our studies, it seems that the number of seeds per capsule may be primarily affected by the characteristics of the habitat. Trepl [45] also refers to the effect of habitat, when he discusses that the seed yield per individual ranges from a few single-seeded capsules to 2000 seeds per individual. In our case, the existence of a site effect is also suggested by the correlation shown by linear regression, in which taller plants were associated with a higher seed number, since taller plant size is generally an indicator of a more favorable habitat. However, the picture is complicated by the fact that rust infection can also cause taller growth, and the quality of the habitat (e.g., its humidity) may also play a role in the development and spread of the infection. Nevertheless, to establish more precise relationships, i.e., to gain a detailed understanding of the relationships between the growing site, fungal infection, plant height and seed number, further studies are necessary, involving more *I. parviflora* stands and supplemented by instrumental measurements of habitat variables.

Regarding seed mass, our results show that both rust and aphids significantly reduced the average seed mass and, thus, the largest seeds were found in the healthy stand. This means that pest- or disease-affected *I. parviflora* plants are generally less able to provide their seedlings with reserve nutrients, which ultimately results in a reduced chance of survival. In the case of *Senecio vulgaris*, which is also an annual weed, the effect of aphids on reducing seed size and also the germinability of seedlings has already been experimentally proven [46]. The average seed mass of the five examined stands damaged by aphids was not the same within the group, as in three stands, a significantly lower seed mass was found compared to the other two stands. This suggests that the seed quality of the stands can be influenced not only by the presence of pests, but also by the characteristics of the growing site. Although the seed size of plant species is generally considered a rather conservative trait, the quality of the growing site can have a significant influence on seed size, as has been demonstrated by several case studies [47,48,49], and the phenomenon was also discussed in detail in the comprehensive book by Fenner and Thompson [50].

As for the applicability of the disease and pest examined as biological control agents, both reduced seed quality, but did not affect seed quantity. The rust fungus in principle could be a promising candidate due to its high host specificity. Besides small balsam, *P. komarovii* has so far only been detected on the Himalayan balsam (*I. glandulifera*), but the latter balsam species is affected by a separate variant, *P. komarovii* var. *glanduliferae*, described in 2015 [24]. In addition to the host specificity of the rust fungus, its negative impact on the seed mass of the host plant also deserves attention. Seed mass is an important fitness component, but not the only one, and seed-mass reduction alone may or may not translate into meaningful demographic impact. Therefore, in future studies, additional demographic traits should also be investigated, such as the germination success of seeds produced by rust-infected individuals. Although the herbivore aphid *I. asiaticum* also resulted in a reduction in the seed mass of small balsam, the main hindrance of its applicability as a biological control agent is its lack of strict host specificity. It attacks *I. parviflora* and *I. glandulifera* in Hungary [21,51], but it has also been reported in *I. noli-tangere* [52] and some further *Impatiens* species [53]. Finally, it is worth mentioning that *I. parviflora* has several other pests besides rust fungus and aphids [51,52], although the usefulness of these pests in biological control against the invasive host plant has not yet been demonstrated.

## 4. Materials and Methods

### 4.1. Study Sites and Plant Materials

To investigate the impact of rust and aphids, we surveyed nine *I. parviflora* stands. Eight stands were located in Hungary and one in southern Poland (Table 3). Two of the surveyed stands were damaged by the rust fungus *P. komarovii* and five by the aphid *I. asiaticum*. We did not find a stand where both pests had simultaneously caused mass colonization. Although occasional aphids may have been present on some rust-infected plants, no mixed infestation was sufficiently abundant to justify its inclusion as a separate category.

**Figure 7:**
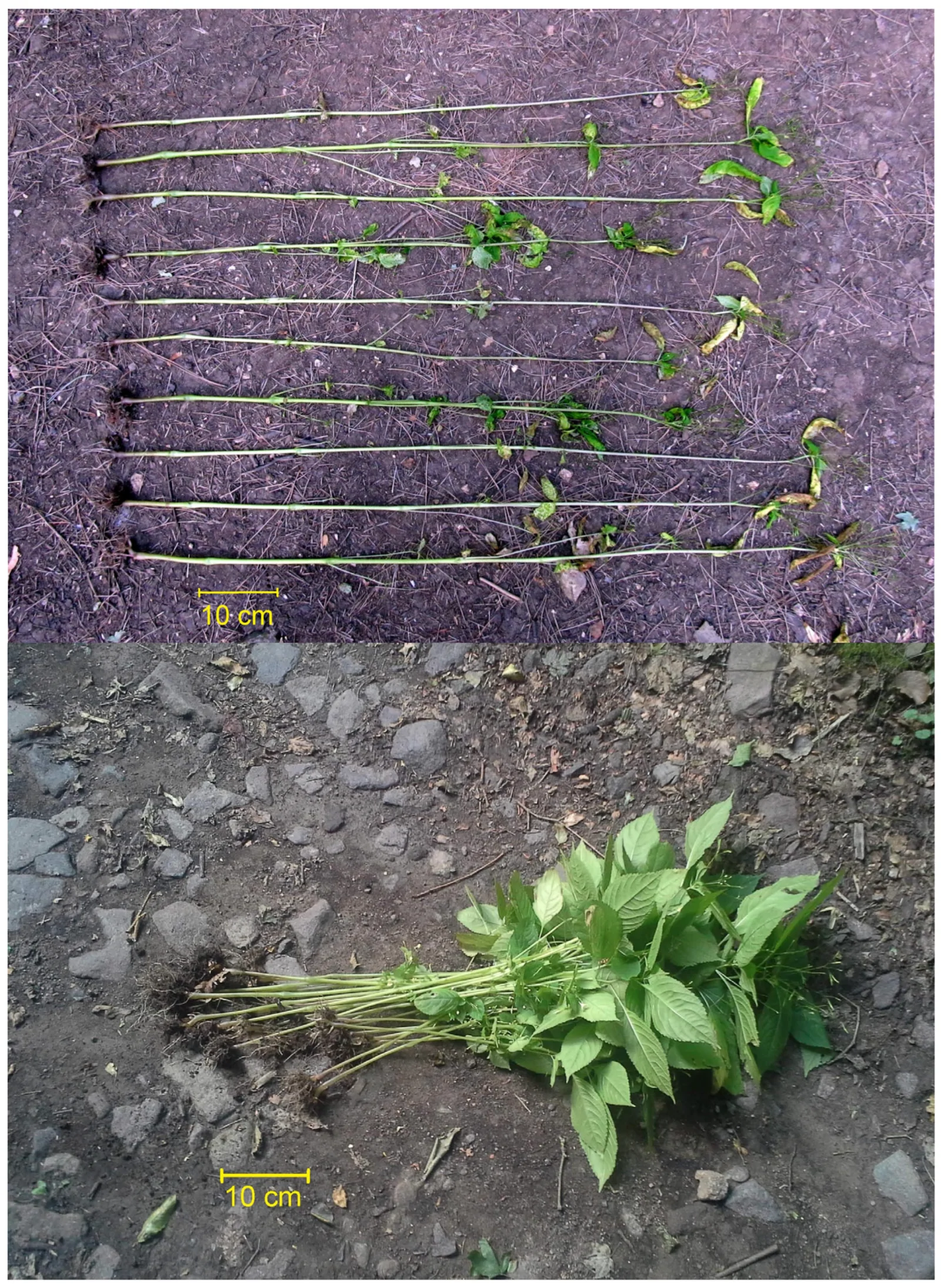
Small balsam specimens infected by *Puccinia komarovii* in old beech stand (R1) at Normafa, Buda Mts., Hungary (**upper photo**); and healthy specimens of small balsam collected in a sessile oak–hornbeam forest at Nagybörzsöny (H2), Börzsöny Mts., Hungary (**lower photo**) (photos by P. Csontos).

The rust fungus was widespread in the two infected stands and was usually observed on the majority of the leaves of each individual of the host plant (Figure 7). In the five stands attacked by aphids, the pests also occurred in large numbers, practically on all host plant specimens, with usually 100–200 individuals damaging each plant. The aphid *I. asiaticum* was identified by an entomology specialist. Two additional stands were free from pests (Figure 7).

### 4.2. Field Sampling and Data Collection

In the inner area of each stand free from edge effects, 20 individuals were randomly selected, which were removed from the soil, and their shoot height above the ground was immediately measured with centimeter accuracy to avoid stem-bending due to desiccation of specimens. Subsequently, 60 mature capsules were selected from 60 additional specimens from the inner area of each stand and the number of mature seeds in them was determined, the maximum value of which, typical for the species, can be 5 seeds per capsule [29]. The seeds found in the capsules were collected for seed weight measurement and were kept in paper bags in a refrigerator at +5 °C. The number of seeds from 60 capsules per stand varied between 93 and 140. Since some seeds were found to be damaged or diseased, the weight of 70 completely healthy seeds per stand were measured on a digital analytical balance (Sartorius R180D) with a readability of 0.01 mg.

### 4.3. Data Processing and Statistical Analysis

The height data of the individuals were analyzed in two ways: (i) first, the nine stands were considered separately and compared with each other; (ii) then, individuals of the same type of stand were combined, and the resulting three groups (i.e., rust-infected, aphid-infected and healthy) were analyzed using statistical methods. In case (i) the raw data had a Gaussian distribution in eight samples, but one stand (H2) did not meet this requirement according to the Kolmogorov–Smirnov test. In addition, according to the Bartlett statistic, there were significant differences between the standard deviations of the individual samples. In case (ii) the Gaussian distribution was again not satisfied for all three data sets. Therefore, in both cases the non-parametric Kruskal–Wallis test was used to evaluate the data. The post hoc test was Dunn’s test with Bonferroni correction in the first case and Dunn’s test in the second.

The graphical interpretation of the seed number per capsule data of the stands is presented in three groups (1-seeded, 2-seeded, and 3-or-more-seeded); however, for the statistical comparison of the individual stands, the basic data were used, which were analyzed with the Kruskal–Wallis test and subsequent Dunn’s test. The relationship between plant height and seed number per capsule was examined using linear regression, and linearity was checked using the Wald–Wolfowitz runs test.

In the case of the seed mass data analysis, we could only consider data from eight stands, as seed mass measurement data were not available for the H2 stand. The comparison of the stands was again carried out using the Kruskal–Wallis test and subsequent Dunn’s test (although data showed Gaussian distribution in all samples, the standard deviations of the samples differed significantly according to Bartlett’s test).

Significant differences were accepted in all cases at *p* < 0.05. InStat ver. 3.06 [54] and PAST ver. 4.03 [55] software packages were used to perform the calculations.

## 5. Conclusions

The impact of pests on the studied plant traits was not uniform. Rust infection increased the height of the host plant. The individuals of the aphid-damaged stands were also taller than those of the healthy stands, although to a lesser extent. In the latter case, the large differences among the studied stands indicate that the effect of the growing location also played an important role. Neither of the two pests tested had a clearly detectable effect on the number of seeds per capsule, but instead a positive correlation was found between the height of the individuals and the number of seeds per capsule. As for the seed weight, both pests significantly reduced the average seed mass in the affected stands, which may be important for biological control against the host plant known as an invasive species. In particular, the rust fungus *P. komarovii* can be considered promising for use in biological plant protection because it shows very high host specificity, but the usefulness of the aphid *I. asiaticum* is highly questionable due to the lack of host specificity.

## Figures and Tables

**Table 1:** Statistical comparison of plant height (cm) data of healthy, rust-infected and aphid-damaged *Impatiens parviflora* stands. Data from stands with the same health status are combined. Results of the Kruskal–Wallis test (a) and Dunn’s test (b). *: *p* < 0.05; **: *p* < 0.01; ***: *p* < 0.001.

**(a) Kruskal–Wallis Test**						
Health Category	Number of Individuals	Minimum Median Maximum			Sum of Ranks	Mean of Ranks
Rust-infected	40	41	72.5	110	4617.0	115.43
Aphid-damaged	100	25	65.0	126	9208.0	92.080
Healthy	40	29	49.5	80	2465.0	61.625
**(b) Dunn’s Test**						
Group 1	Group 2	Difference in Rank Means			*p*-Value	Conventional Notation
Rust-infected	Aphid-damaged	23.345			*p* = 0.0166	*
Rust-infected	Healthy	53.800			*p* = 3.863 × 10^−6^	***
Aphid-damaged	Healthy	30.455			*p* = 0.00178	**

**Table 2:** Results of pairwise comparisons of nine *Impatiens parviflora* stands based on the number of seeds per capsule data examined in 60 capsules per stand. The statistical evaluation was based on the Kruskal–Wallis test followed by Dunn’s test. R1–2: infected by rust fungus *Puccinia komarovii*; L1–5: damaged by aphid *Impatientinum asiaticum*; H1–2: healthy stands; ns: not significant; *: *p* < 0.05; ***: *p* < 0.001 (see Section 4.1 for more information about stands).

Stand Pairs Compared	Rank-Mean Difference	Level of Significance	*p*-Value
R1 vs. R2	−19.800	ns	*p* > 0.05
R1 vs. L1	17.675	ns	*p* > 0.05
R1 vs. L2	−57.108	ns	*p* > 0.05
R1 vs. L3	13.592	ns	*p* > 0.05
R1 vs. L4	31.475	ns	*p* > 0.05
R1 vs. L5	−109.630	***	*p* < 0.001
R1 vs. H1	33.183	ns	*p* > 0.05
R1 vs. H2	34.058	ns	*p* > 0.05
R2 vs. L1	37.475	ns	*p* > 0.05
R2 vs. L2	−37.308	ns	*p* > 0.05
R2 vs. L3	33.392	ns	*p* > 0.05
R2 vs. L4	51.275	ns	*p* > 0.05
R2 vs. L5	−89.825	*	*p* < 0.05
R2 vs. H1	52.983	ns	*p* > 0.05
R2 vs. H2	53.858	ns	*p* > 0.05
L1 vs. L2	−74.783	ns	*p* > 0.05
L1 vs. L3	−4.083	ns	*p* > 0.05
L1 vs. L4	13.800	ns	*p* > 0.05
L1 vs. L5	−127.300	***	*p* < 0.001
L1 vs. H1	15.508	ns	*p* > 0.05
L1 vs. H2	16.383	ns	*p* > 0.05
L2 vs. L3	70.700	ns	*p* > 0.05
L2 vs. L4	88.583	*	*p* < 0.05
L2 vs. L5	−52.517	ns	*p* > 0.05
L2 vs. H1	90.292	*	*p* < 0.05
L2 vs. H2	91.167	*	*p* < 0.05
L3 vs. L4	17.883	ns	*p* > 0.05
L3 vs. L5	−123.220	***	*p* < 0.001
L3 vs. H1	19.592	ns	*p* > 0.05
L3 vs. H2	20.467	ns	*p* > 0.05
L4 vs. L5	−141.100	***	*p* < 0.001
L4 vs. H1	1.708	ns	*p* > 0.05
L4 vs. H2	2.583	ns	*p* > 0.05
L5 vs. H1	142.810	***	*p* < 0.001
L5 vs. H2	143.680	***	*p* < 0.001
H1 vs. H2	0.875	ns	*p* > 0.05

**Table 3:** Locations and site characteristics of the sampled *Impatiens parviflora* stands investigated for plant size and reproductive traits. R = infected by rust fungus *Puccinia komarovii*; L = damaged by aphid *Impatientinum asiaticum*; H = healthy stand.

Code	Geographical Location Vegetation Type	GPS Coordinates	Altitude Above Sea Level	Mean Annual Precipitation	Health Status	Sampling Date (yyyy.mm.dd)
R1	Normafa, Budai Mts., Hungary; old beech stand	47°29′57.7″ N 18°58′10.1″ E	460 m	615 mm	R	2013.07.20.
R2	Hárs Mt., Budai Mts., Hungary; oak stand	47°31′44″ N 18°57′22.6″ E	390 m	605 mm	R	2013.07.23.
L1	Denevér street, Budapest, Hungary; ash grove at valley bottom	47°29′35.8″ N 18°59′34.9″ E	327 m	600 mm	L	2013.07.26.
L2	Normafa, Budai Mts., Hungary; gap in a mixed stand of beech and sessile oak	47°30′8.4″ N 18°57′54.6″ E	490 m	610 mm	L	2013.07.29.
L3	Kis-Sváb Hill, Budai Mts., Hungary; mixed forest of lime and ash on slopes	47°30′17.5″ N 19°00′40.9″ E	197 m	595 mm	L	2013.07.26.
L4	Near Budakeszi, Budai Mts., Hungary; sessile oak–hornbeam stand	47°30′25.4″ N 18°56′38.4″ E	320 m	620 mm	L	2013.07.27.
L5	Near Bielsko-Biała, Poland; mixed deciduous forest	49°47′43″ N 19°06′48.8″ E	450 m	890 mm	L	2013.09.09.
H1	Vidra farm, Vámosszabadi, Hungary; riverine willow–poplar woodlands	47°47′5.4″ N 17°37′22.6″ E	115 m	645 mm	H	2013.08.26.
H2	Nagybörzsöny, Börzsöny Mts., Hungary; sessile oak–hornbeam forest	47°55′42.6″ N 18°50′36.3″ E	270 m	690 mm	H	2016.07.21.

## Data Availability

The raw data supporting the conclusions of this article will be made available by the authors on request.
